# Cytotoxicity and Binding to DNA, Lysozyme, Ribonuclease A, and Human Serum Albumin of the Diiodido Analog of Picoplatin

**DOI:** 10.1021/acs.inorgchem.4c05424

**Published:** 2025-05-02

**Authors:** Giarita Ferraro, Jitka Pracharova, Giovanni Gotte, Lara Massai, Michal Berecka, Pavel Starha, Luigi Messori, Antonello Merlino

**Affiliations:** † Department of Chemical Sciences, University of Naples Federico II, Complesso Universitario di Monte Sant’Angelo, via Cinthia 21, Naples 80126, Italy; ‡ Department of Biophysics, Faculty of Science, Palacký University Olomouc, Slechtitelu 27, Olomouc 783 71, Czech Republic; § Department of Neuroscience, Biomedicine, and Movement Sciences, Biological Chemistry Section, University of Verona, Strada Le Grazie 8, Verona I-37134, Italy; ∥ Department of Chemistry “Ugo Schiff”, University of Florence, via della Lastruccia 3–13, Sesto Fiorentino 50019, Florence, Italy; ⊥ Department of Inorganic Chemistry, Faculty of Science, Palacký University Olomouc, 17 listopadu 1192/12, Olomouc 771 46, Czech Republic

## Abstract

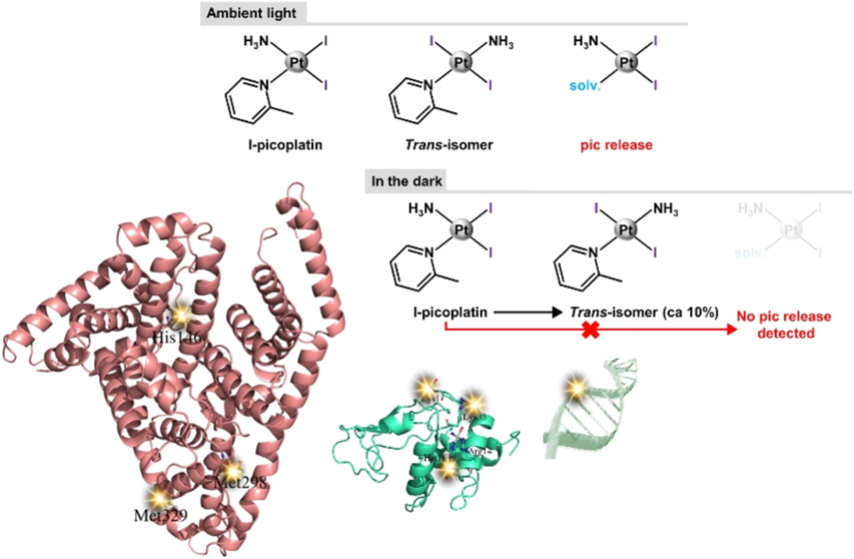

Here we investigated cytotoxicity and DNA and protein binding of an iodido analog of picoplatin, the *cis*-ammine-diiodido(2-methylpyridine)platinum(II) complex (I-picoplatin). I-picoplatin (IC_50_ = 3.7–12.4 μM) outperforms picoplatin (IC_50_ = 11.8–22.6 μM) in the human cancer cell lines used and shows a greater ability to overcome the cisplatin resistance of A2780 ovarian cancer cells than does picoplatin. I-picoplatin also induces different cell cycle changes (reduced S-phase fraction and an increase in the G2/M phase arrest) in HeLa cervical carcinoma cells compared to both picoplatin and cisplatin. Binding of the metal compound to DNA model systems was investigated by ethidium bromide displacement assay and circular dichroism. Its reactivity with lysozyme (HEWL) and pancreatic RNase A was studied by X-ray diffraction and mass spectrometry experiments. I-picoplatin binds the DNA double helix and is able to retain the 2-methylpyridine ligand and at least one of the two iodido ligands when bound to the two proteins. Various Pt-containing moieties, including one based on the isomerized structure of I-picoplatin, coordinate the His and Met residues. A low-resolution structure of the I-picoplatin/human serum albumin (HSA) adduct has also been solved. The side chains of His146, Met289, and Met329 are the primary binding sites of the I-picoplatin moieties on HSA.

## Abstract 2

Cytotoxicity and binding to protein and DNA of the *cis*-ammine-diiodido(2-methylpyridine)platinum(II) complex (I-picoplatin) were investigated.

## Introduction

**Figure 1:**
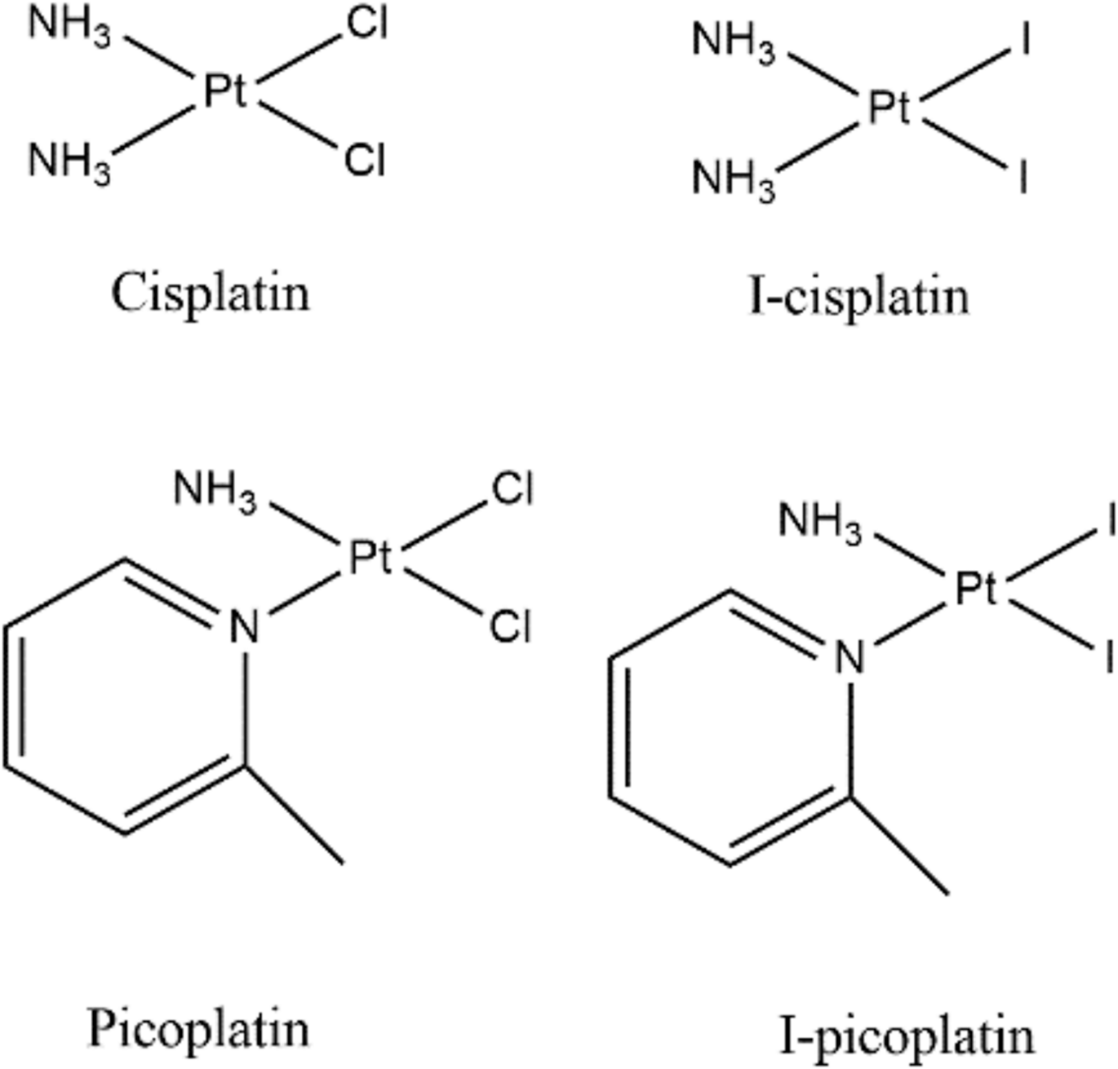
Structures of cisplatin, picoplatin, and their iodinated derivatives.

Cisplatin (Figure 1), carboplatin, and oxaliplatin are used to treat many solid tumors, either alone or in combination with other anticancer agents.^1−3^ According to the United States’ National Cancer Institute (NCI) database, 10–20% of all cancer patients are treated with these Pt-based drugs (https://www.cancer.gov/research/progress/discovery/cisplatin).^4^ However, the use of these compounds is associated with the emergence of several side effects and resistance phenomena,^5^ and, therefore, several new Pt-based anticancer agents have been synthesized and characterized in recent decades with the aim of overcoming these limitations.^6−9^ Picoplatin ((*cis*-amminedichlorido[2-methylpyridine]platinum[II]), Figure 1) is a Pt-based drug with proven anticancer activity *in vitro*(10) and *in vivo*.^11^ It has been shown to be active against small-cell lung cancer^10−13^ and is effective against cancer cells that are resistant to cisplatin and the other Pt-based drugs.^10−13^

The binding of Pt-based drugs to DNA is recognized as a crucial event for the anticancer activity of these molecules.^14^ Binding induces DNA damage that ultimately leads to cancer cell death. However, DNA is not the only target of these drugs, which can also bind proteins.^15,16^ Protein platination affects drug bioavailability and toxicity and plays an important role in the mechanism of action of anticancer Pt complexes.^17,18^ We have studied the platination process of several proteins using a combined crystallographic/electrospray ionization mass spectrometry (ESI-MS) approach.^19,20^ The structures of the adducts of cisplatin,^21−25^ its diiodido analog^26^ and picoplatin^27^, upon reaction with hen egg white lysozyme (HEWL) and pancreatic RNase A have been reported.

Cisplatin binds HEWL by coordinating the side chains of His15 or both Arg14 and His15 after the release of a chloride ligand^21,23,25^ and RNase A at the level of the side chains of Gln28/Met29, His105 and His119.^22,24^ Both iodide ligands remain bound to the metal in the adduct formed when HEWL reacts with *cis*-[PtI_2_(NH_3_)_2_].^26^

In the case of picoplatin, results indicate that Pt fragments are bound to His15, Asp18, Asp119, and both Lys1 and Glu7 of HEWL, and to His12, Met29, His48, Asp53, Met79, His105, and His119 of RNase A, without affecting the overall structure of the two enzymes, but possibly altering the metal binding site environment.^27^

A diiodido analog of picoplatin (I-picoplatin) has been recently synthesized and characterized (Figure 1).^28^ Surprisingly, this compound is unstable when exposed to light in solution, where it releases its 2-methylpyridine (pic) ligand and isomerizes.^28^

Here we studied the cytotoxicity of I-picoplatin against human ovarian carcinoma A2780 (parent cisplatin-sensitive) and A2780R (cisplatin-resistant) cells, cervical carcinoma (HeLa), *esophageal* carcinoma (OE33), triple-negative breast carcinoma (MDA-MB-231) cell lines, and normal human lung tissue cells (MRC-5 pd30). Furthermore, to gain insight into the interaction of this compound with biological macromolecules and to compare its reactivity with proteins with that of picoplatin,^27^ cisplatin^21,25,29−31^ and the diiodido analog of cisplatin^26^ (Figure 1), DNA binding assays have been carried out and the molecular structures of the adducts formed with HEWL and RNase A, and ESI-MS data on the same adducts have been collected. Furthermore, we have determined a low-resolution structure of the adduct that I-picoplatin forms with human serum albumin. The results of this structural determination have been compared with those obtained when crystals of HSA were treated with cisplatin.^32,33^

## Experimental Section

### Synthesis and NMR Stability Studies

I-picoplatin and picoplatin were prepared according to the literature.^28^ Solvents for NMR experiments (DMF-*d*_7_, D_2_O) were purchased from Chemstar (Pilsen, Czech Republic).

I-picoplatin was dissolved in 600 μL of DMF-*d*_7_, and ^1^H NMR spectra were acquired at *t* = 0–24 h. The sample was protected from light during the individual experiments. ^1^H NMR spectrum of α-picoline in DMF-*d*_7_ was also acquired for comparison.

### Cell Lines and Culture

The human ovarian carcinoma A2780 (parent cisplatin-sensitive) and A2780R (cisplatin-resistant) cells were provided by Prof. B. Keppler, University of Vienna (Austria). The human cervical carcinoma HeLa, the triple-negative human breast carcinoma MDA-MB-231, the esophageal carcinoma OE33 cell lines, and the human normal lung tissue MRC-5 pd30 cells were obtained from the European Collection of Authenticated Cell Cultures (ECACC, Salisbury, U.K.). The OE33, A2780, and A2780R were cultured in RPMI 1640 medium (Biosera, Boussens, France) supplemented with streptomycin (100 μg mL^–1^, Sigma, Prague, Czech Republic), penicillin (100 U mL^–1^, Sigma, Prague, Czech Republic), and 10% fetal bovine serum (FBS, PAA, Pasching, Austria), which was heat-inactivated at 56 °C before use. The acquired resistance of A2780R cells was preserved by adding cisplatin (1 μM; Sigma, Prague, Czech Republic) to the medium every second subculture. The HeLa, MDA-MB-231, and MRC-5 pd30 cells were cultured in DMEM (high glucose, 4.5 g L^–1^, Biosera, Boussens, France) supplemented with streptomycin (100 μg mL^–1^), penicillin (100 U mL^–1^), and FBS, which was heat-inactivated at 56 °C before use. The cells were maintained in a humidified incubator at 37 °C under a 5% CO_2_ atmosphere and subcultured twice weekly with an appropriate plating density.

### Antiproliferative Activity Testing

To evaluate the effect of platinum complexes on cell viability, the colorimetric MTT assay was used. Cells were plated in 96-well tissue culture plates (TPP, Switzerland) at a density of 10^4^ cells/well for A2780, A2780R, and OE33, respectively, 5 × 10^5^ cells/well for HeLa, MDA-MB-231, and MRC-5 pd30 in 100 μL of growth medium and incubated at 37 °C in a humidified 5% CO_2_ atmosphere for 16 h (overnight). After incubation, the cells were exposed to Pt complexes and maintained in the incubator for additional 72 h. The stock solutions of Pt compounds were always prepared in DMF (Serva, Heidelberg, Germany) prior to use. The final concentration of DMF in the cell culture medium did not exceed 0.1% (v/v). This concentration has no effect on the cell growth. Later, 10 μL of MTT solution (5 mg mL^–1^; Calbiochem, Darmstadt, Germany) was added to each well, and the plates were kept under the cultivation conditions for 4 h. At the end of the incubation, the medium was discarded. Then, the formazan product was solubilized in 100 μL of DMSO per well (Serva, Heidelberg, Germany). Cell viability was assessed by measuring the absorbance at 570 nm (reference wavelength at 630 nm) using a Spark Tecan Schoeller reader. The reading values were transformed into a percentage of control (% cell survival). From curves constructed by plotting cell survival (%) versus drug concentration (μM), the IC_50_ values were calculated. Antiproliferative effects were expressed as the IC_50_ from three independent experiments. The IC_50_ value is defined as the concentration of the agent that inhibits cell growth by 50%.

### Cell Cycle Perturbation Studies

Confluent HeLa cells were seeded at a density of 5 × 10^5^ cells/well in six-well culture plates (TPP, Switzerland). The cells were then kept in a drug-free medium under cultivation conditions overnight. Subsequently, the cells were treated with the Pt compounds at their final concentrations corresponding to IC_50,72 h_ values. The treatment period was 24 h. Then, the cells were trypsinized, washed twice with PBS, and fixed using 70% ethanol at 4 °C (Serva (Heidelberg, Germany)). Fixed cells were processed as follows: propidium iodide staining (50 μg mL^–1^; Sigma (Prague, Czech Republic)) in Vindel’s solution (10 mM Tris-Cl, pH 8.0, 10 mM NaCl, 0.1% Triton X-100, 100 μg mL^–1^ RNase A) for 30 min at 37 °C was performed. After the staining, samples were measured with a FACS Verse flow cytometer (Becton Dickinson, Germany), and cell cycle profiles were obtained. Data were processed in FCS Express (DeNovo Software, CA). All experiments were performed in triplicate.

### DNA-Binding Studies

Calf thymus double-strand DNA (ct-DNA, Sigma-Aldrich) solution (500 μM) was prepared by dissolving the solid sample into 50 mM ammonium acetate (AMAC) pH 7.5. Ethidium bromide (EB) was purchased from Sigma-Aldrich and used without further purification.

A fluorescence quenching assay was performed using a JASCO FP 8300 spectrofluorometer equipped with a thermostat bath using 1.0 cm quartz cells. Ct-DNA was diluted in the buffer and treated with EB at a DNA:EB molar ratio of 5:1 for 30 min in the dark. It has been verified that EB binds to DNA and that DMSO does not affect the DNA–EB interaction. The quenching was evaluated by adding 2.0 μL of the Pt complex solution, obtained by dissolving the compound in DMSO (30 mM), to the DNA–EB complex solution, following the fluorescence intensity after the addition of Pt-compound upon excitation at 545 nm. Other experimental settings were 5.0 nm excitation/emission slit, 1.0 nm data pitch, emission range 560–750 nm, and equilibration time 5 min.

Circular dichroism (CD) spectra of ct-DNA untreated and treated with I-picoplatin were recorded at 25 °C using a Jasco J-810 spectropolarimeter. CD spectra of 200 μM ct-DNA in 10 mM AMAC, pH 7.5, were registered in the range of 260–350 nm, averaging three scans for each measurement and using a 0.1 cm quartz cuvette. Ct-DNA was incubated with different concentrations of I-picoplatin for 72 h to reach the following ct-DNA:Pt molar ratios: 1:0.5, 1:1, 1:2, and 1:3. Other analysis settings were scanning speed 50 nm × min^–1^, resolution 0.2 nm, sensitivity 50 mdeg, response 2 s, bandwidth 2.0 nm.

### ESI-MS Experiments

#### ESI-MS Analysis Settings

One mM stock solutions (1 mM) of RNase A and HSA (both from Merck) were prepared by solubilizing the respective lyophilized proteins in LC-MS grade water. No further purification was performed before use. Ten mM stock solutions of I-picoplatin were prepared by solubilizing the sample in DMSO. For each experiment, a 1:3 molar ratio of protein stock solution and platinum compound was diluted in 2 mM ammonium acetate (pH 6.8) containing 3% DMSO to achieve a final protein concentration of 100 μM.

The mixtures were incubated at 37 °C for up to 48 h. Following incubation, samples were diluted to a final protein concentration of 500 nM in 2 mM ammonium acetate solution, pH 6.8, with 0.1% (v/v) formic acid immediately added prior to mass spectrometry analysis.

Instrumental parameters: direct infusion in ESI-MS was performed at a 7 μL min-1 flow rate in a TripleTOF 5600+ high-resolution mass spectrometer (Sciex, Framingham, MA), equipped with a DuoSpray interface operating with an ESI probe.

For each protein, ESI source parameters were optimized as follows.

RNase A: positive polarity, ion spray voltage floating (ISFV) 5500 V, temperature (TEM) 0, gas 1 (GS1) 40 L/min; gas 2 (GS2) 0; curtain gas (CUR) 15 L/min, declustering potential (DP) 100 V, collision energy (CE) 10 V; range 1000–2600 *m*/*z*.

HEWL: positive polarity, ISFV 5500 V, TEM 0, GS1 40 L/min; GS2 0; CUR 20 L/min, DP 100 V, CE 10 V; range 1000–2800 *m*/*z*.

#### Crystallization of the Adducts Formed by I-Picoplatin with HEWL and RNase A

HEWL and RNase A (XIIA) from Sigma-Aldrich were used without further purification. I-picoplatin has been synthesized according to the procedure previously described.^28^ Crystals of the adduct of the metal complex with the two proteins have been obtained by a soaking procedure,^26^ by treating crystals of the two proteins with a saturated solution of the Pt-compound. Crystals of metal-free proteins were grown using hanging drop vapor diffusion. One μL of protein and 1 μL of the reservoir were mixed in the drop. HEWL crystals were grown using a protein concentration of 15 mg mL^–1^ and 1.1 M NaCl and 0.1 M sodium acetate at pH 4.5 for structure A, 0.8 M succinic acid at pH 7.0 for structure B, 2.0 M sodium formate, and 0.1 Hepes buffer pH 7.5 for structure C. RNase A crystals were grown using a protein concentration of 22 mg mL^–1^ and 20% PEG4000, 0.1 M sodium citrate pH 5.1. Crystals were exposed to I-picoplatin solution for 3 and 7 days in the case of HEWL and RNase A, respectively.

#### Crystallization of the I-Picoplatin/HSA Adduct

HSA was purchased from Merck and extensively purified before crystallization trials. To purify the protein sample from its oligomeric forms, a Superdex 200 HL 16/60 prep grade SEC column connected with an ÄKTA Purifier chromatograph (Cytiva, Milan, Italy) was used. The column was equilibrated with 25 mM Tris pH 7.4, 100 mM NaCl, and the same buffer was used as a running buffer at 1.0 mL min^–1^ flow rate. HSA was identified by SDS PAGE and concentrated up to more than 100 mg mL^–1^ with Amicon Plus 10 kDa c.off ultrafilters (Merck-Millipore) in ddH**_2_**O.

Crystals of the adduct of I-picoplatin with HSA have been obtained by a soaking procedure, following the protocol used to obtain crystals of the adduct of HSA with cisplatin.^32^ Briefly, HSA crystals were grown by the sitting drop vapor diffusion method using equal volumes of 100 mg mL^–1^ HSA and of a solution consisting of 25–30% (w/v) PEG3350, 50 mM potassium phosphate, pH 7.5 (reservoir). Then, HSA crystals were soaked for 7 days in a solution of the reservoir, which was previously saturated with I-picoplatin.

#### Data Collection and Refinement

X-ray diffraction data collection on HEWL, RNase A, and HSA crystals treated with I-picoplatin has been carried out on frozen crystals, which have been cryoprotected using a solution of the reservoir containing 30% (v/v) glycerol.

Diffraction data collections have been carried out at the Elettra synchrotron (Trieste, Italy) on the XRD2 beamline. Data have been scaled using the AutoPROC pipeline.^34^ Data collection and refinement statistics are listed in Table S1. The structures have been solved by molecular replacement using Phaser.^35^ Coordinates from PDB codes 1JVT,^36^193L,^37^ and 4S1Y,^32^, without water and ligands, have been used as starting models. Restrained refinement has been performed using Refmac5.^38^ Since the diffraction data collected on crystals of the I-picoplatin/HSA adduct were limited to 3.9 and 4.2 Å, low-resolution refinements using ProSMART (Procrustes Structural Matching Alignment and Restraints Tool)^39^ to generate hydrogen bond external restraints (e.g., secondary structure restraints) were carried out for this structure. Inspection of electron density (e.d.) maps and model building have been carried out using Coot.^40^

To identify Pt centers, anomalous difference e. d. maps have been inspected. Pt occupancy has been evaluated by minimizing residual Fourier difference e. d. maps peaks.

PDB validation server (www.rcsb.org) and Coot routines^40^ were used to validate the structures, which were deposited in the PDB with these codes: 9HLK, 9HMK, 9HMQ, 9HN6, 9HNB. Pymol (www.pymol.org) was used to produce the figures.

## Results and Discussion

### Synthesis and Stability

**Scheme 1:**
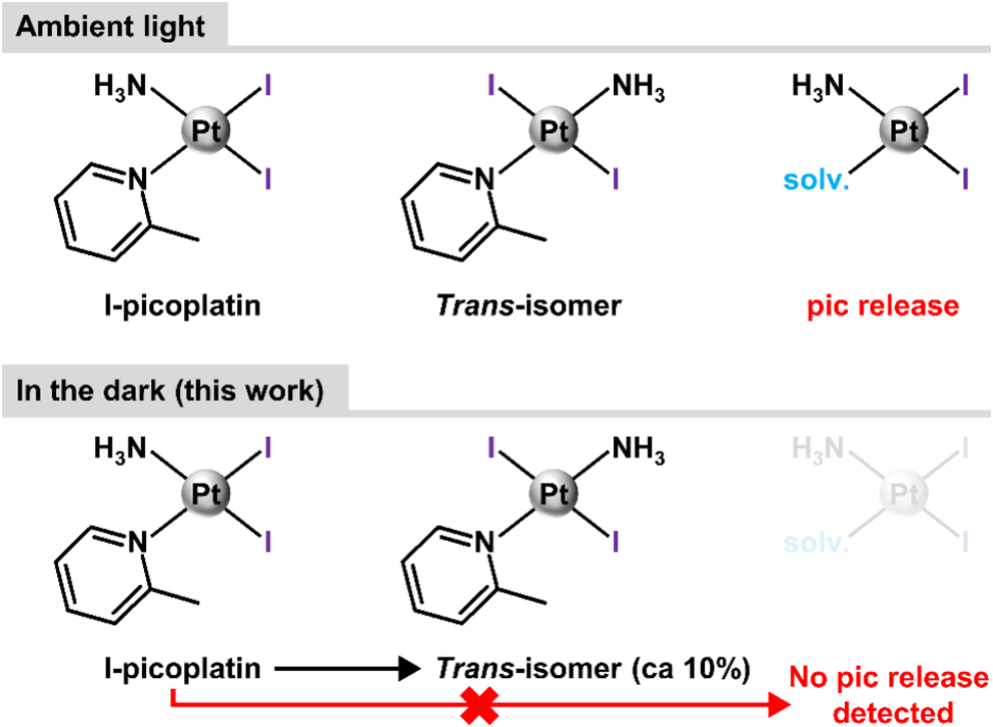
Pt-Based Species Detected in DMF Solution of I-Picoplatin by ^1^H NMR The studied solutions were kept under ambient light (top, [ref (28)]) or in the dark (bottom; this work) between the individual experiments recorded at different time points (0–24 h).

Picoplatin and I-picoplatin (Figure 1) were prepared as previously described.^28^ It has recently been reported that I-picoplatin is unstable in DMF and in an aqueous solution (i.e., 50% DMF-*d*_7_/50% D_2_O) when exposed to ambient light (Scheme 1). In particular, a release of the pic ligand was observed. For this reason, its stability was studied by ^1^H NMR in DMF (to avoid hydrolysis) in the dark. This experiment is motivated by our interest in the investigation of I-picoplatin *in vitro* antiproliferative activity and processes connected with its mechanism of antiproliferative action (i.e., interaction with proteins). Simply said, if I-picoplatin were unstable even in the dark, it would have to be excluded from the intended antiproliferative activity experiments.

However, the obtained ^1^H NMR results showed that I-picoplatin is stable when protected from light, making it suitable for biological experiments. No release of the pic ligand was observed in the dark (Scheme 1). The only change of I-picoplatin is connected with its partial *cis*-to-*trans* isomerization, as discussed in our previous work.^28^ Approximately 10% of I-picoplatin changed to the *trans*-isomer (new resonances at δ = 9.04, 7.83, 7.31, and 3.14 ppm) after 24 h (Figure S1).

### *In Vitro* Antiproliferative Activity

Since numerous Pt(II) diiodido complexes outperform their dichlorido congeners in terms of *in vitro* antiproliferative activity, we hypothesized that the potency of I-picoplatin could exceed picoplatin; thus, it could be of pharmacological interest.

The antiproliferative activity of I-picoplatin (picoplatin and cisplatin were also studied for comparative purposes) was investigated in A2780, A2780R, HeLa, OE33, and MDA-MB-231 cancer cell lines. The testing was also conducted with healthy lung fibroblast MRC-5 pd30. The results of *in vitro* antiproliferative activity testing (given as IC_50_ [μM]) are summarized in Table 1 (72 h exposure time).

**Table 1:** *In Vitro* Antiproliferative Activity (IC_50_ ± SD; μM) of I-Picoplatin, Picoplatin, and Cisplatin in Human Ovarian Carcinoma A2780 (Parent Cisplatin-Sensitive) and A2780R (Cisplatin-Resistant) Cells, Cervical Carcinoma (HeLa), Esophageal Carcinoma (OE33), Triple-Negative Breast Carcinoma (MDA-MB-231) Cell Lines, and Normal Human Lung Tissue MRC-5 pd30 Cells Determined by the MTT Assay (72 h Exposure Time)^a^

cell line	I-picoplatin	picoplatin	cisplatin
A2780	3.7 ± 0.8	11.8 ± 0.8	2.5 ± 0.3
A2780R	4.9 ± 0.3 (1.3)	21.3 ± 0.7 (1.8)	12.8 ± 0.9 (5.1)
HeLa	6.0 ± 1.0	22.6 ± 4.7	17.2 ± 0.9
OE33	5.4 ± 2.2	12.5 ± 0.3	6.7 ± 0.6
MDA-MB-231	12.4 ± 1.6	17.2 ± 1.7	23.4 ± 2.5
MRC-5 pd30	23.2 ± 1.2	31 ± 2.5	12.5 ± 0.2

a The results in the table are means ± SD of three independent tests, each made in quadruplicate. The resistance factor (RF) is given in parentheses. This RF is defined as IC_50_(resistant)/IC_50_(sensitive) cells.

I-picoplatin showed low-micromolar potency in the panel of human cancer cell lines used, with cytotoxicity higher than that observed for picoplatin and cisplatin, except in A2780 cells, where I-picoplatin expressed lower activity than cisplatin. In particular, I-picoplatin was markedly more toxic than both cisplatin and picoplatin in the A2780R cells. The resistance factor is defined as the ratio of IC_50_ values between cisplatin-resistant (A2780R) and -sensitive (A2780) cells. This factor was 5.1 and 1.8 for cisplatin and picoplatin, respectively, while it was significantly lower for I-picoplatin (1.3). Thus, it is confirmed that picoplatin reduces the resistance factor in ovarian cancer cell lines with acquired resistance to cisplatin (A2780 model in this work), consistent with previously published results for different ovarian cancer cells.^41^ More importantly, I-picoplatin has the capacity to overcome the acquired resistance of cancer cells to cisplatin treatment and is more effective than picoplatin.

The comparison between A2780R and A2780 cells is particularly noteworthy for picoplatin and I-picoplatin, as A2780R cells exhibit significantly elevated glutathione (GSH) levels compared to their parental line.^42^ Picoplatin was specifically designed to be less susceptible to inactivation by the substances that contain thiols, such as GSH, which are linked to tumor resistance, compared to cisplatin.^43^ Previous publications have shown that picoplatin can also overcome acquired cisplatin resistance in other cell line pairs (parental/cisplatin-resistant), where elevated GSH levels were not part of the resistance mechanism.^11,41^

I-picoplatin was also more toxic than cisplatin and picoplatin in HeLa and triple-negative MDA-MB-231 cells. Notably, both cell lines are intrinsically resistant to cisplatin treatment. This indicates that the mechanism responsible for the biological action of I-picoplatin differs, to some extent, from that of cisplatin. This difference allows I-picoplatin to successfully overcome the resistance mechanisms operating in the case of cisplatin and picoplatin. Another notable difference includes the lower toxicity of both I-picoplatin and picoplatin in noncancerous lung fibroblast MRC-5 pd30. Thus, these compounds showed better selectivity for tumor cells over normal cells compared to cisplatin. The antiproliferative activity toward the cisplatin-sensitive OE33 cell line for I-picoplatin and cisplatin was almost the same but picoplatin was less effective in killing these cells.

### Cell Cycle Analysis

The results of the antiproliferative activity experiments toward human cancer cells revealed that I-picoplatin exhibits higher activity than picoplatin and cisplatin in the selected lines. The highest activity of I-picoplatin compared with cisplatin and picoplatin was observed for the HeLa cell line. These findings motivated us to study the effect of the selected Pt compounds on the cell cycle of these cells. HeLa cells were then treated with roughly equitoxic concentrations (IC_50_ values found for these compounds in HeLa cells treated for 72 h are shown in Table 1) of the Pt compounds for 24 h. After the treatment period, the cells were collected by trypsinization, ethanol fixed, and stained with propidium iodide. Samples were then processed for cell cycle distribution via flow cytometry.

**Figure 2:**
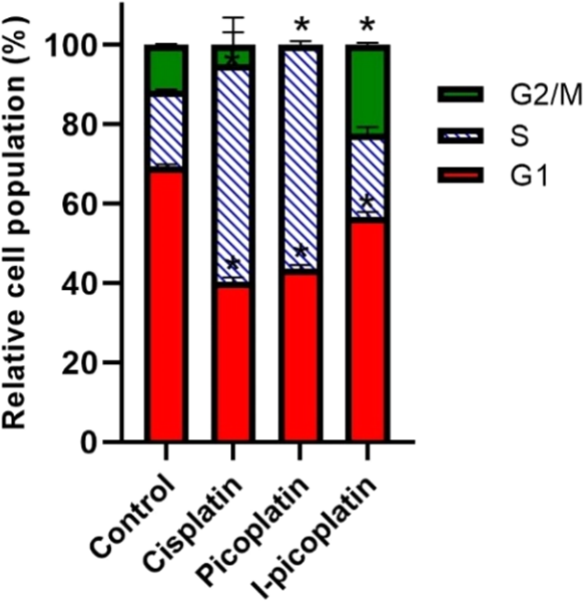
Cell cycle analysis of HeLa cells. Effects of cisplatin, picoplatin, and I-picoplatin on cell cycle distribution after 24 h of treatment with roughly equitoxic concentrations (IC_50_ found for these compounds in HeLa cells treated for 72 h, Table 1). Percentages of counts assigned to the individual populations are color coded as follows: G1 (red), S (blue dashed), and G2/M (green). The estimated percentages of different populations (different cell cycle phases) of HeLa cells were acquired using FCS Express software. The results are expressed as the mean ± SD of three independent tests. Stars at the columns in the graph indicate statistically significant difference compared to control, untreated cells (*p* < 0.01).

As illustrated in Figure 2, cisplatin induces S-phase arrest in the HeLa cell cycle, leading to a decrease in the G2/M phase population. This outcome aligns with the cisplatin DNA-damaging mechanism of action, consistent with previously published data.^43,44^ Similarly, picoplatin also arrests the HeLa cell cycle, preventing progression to the G2/M phase, which is nearly absent. This observation corroborates the reported primary mechanism of action of picoplatin, namely, interference with DNA replication.^45,46^

These findings are further supported by antiproliferative activity tests, which demonstrated comparable efficacy of cisplatin and picoplatin (within the margin of experimental error) in HeLa cells.

In contrast, treatment with I-picoplatin results in a reduced S-phase fraction and a significant increase in the G2/M phase fraction compared to that of cisplatin- and picoplatin-treated samples, indicating G2/M phase arrest. As shown in Figure 2, I-picoplatin exhibits a distinct impact on cell cycle phase distribution under the investigated experimental conditions. Moreover, the antiproliferative activity (Table 1) suggests that I-picoplatin retains biological activity against this resistant cell line.

### Interaction with DNA

The results obtained from testing the selected Pt compounds on the cancer cells suggest that I-picoplatin behaves differently from cisplatin and picoplatin. The interaction of DNA with Pt-based drugs is acknowledged as a key factor in their anticancer efficacy.^14^ Thus, to obtain some insight into the mechanism of action of I-picoplatin, we studied its reactivity with both DNA and model proteins.

**Figure 3:**
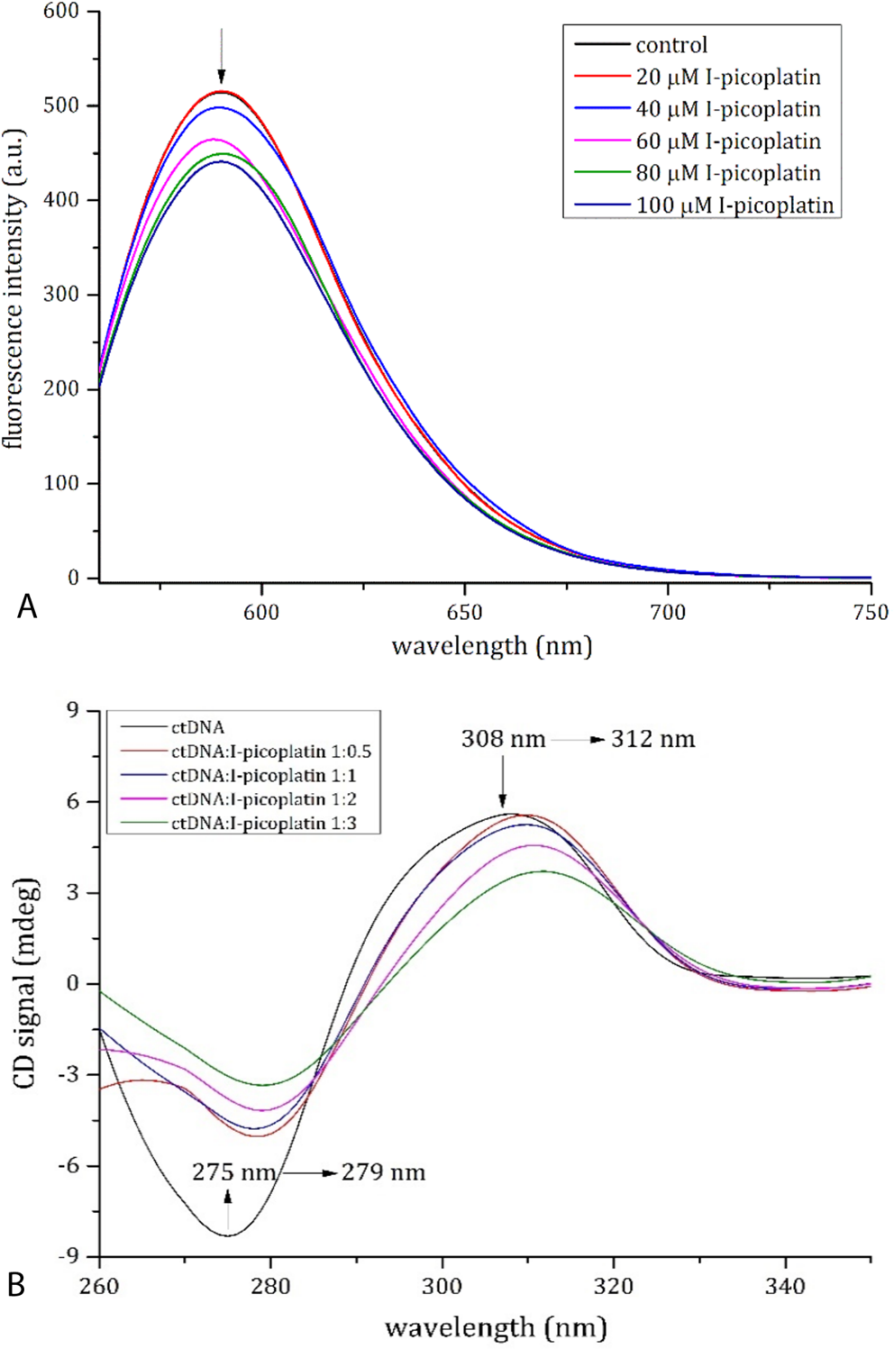
(A) Emission spectra of ct-DNA-ethidium bromide complex in the absence and the presence of I-picoplatin (0–100 μM). (B) CD spectra of ct-DNA (200 μM in 10 mM AMAC, pH 7.5) in the absence (black line) and in the presence of I-picoplatin at 1:0.5 (brown line), 1:1 (blue), 1:2 (violet), and 1:3 (green) ct-DNA to Pt-complex molar ratio.

Binding of I-picoplatin to calf thymus DNA was examined by using the fluorescence assay and CD spectroscopy. In the fluorescence assay, the quenching associated with the displacement of ethidium bromide was used as a probe of the binding of I-picoplatin to the double helix. Indeed, the I-picoplatin binding to the DNA is accompanied by a reduction of the fluorescence intensity due to the displacement of EB from the ct-DNA-EB complex (Figure 3A). This result indicates that I-picoplatin binds the double helix, displacing the chromophore from its position between base pairs. To evaluate if the binding of I-picoplatin to ct-DNA was associated with a change in the double helix conformation, CD spectra of the nucleic acid upon addition of different amounts of the Pt complex were collected. CD profile of ct-DNA slightly changes in the presence of I-picoplatin (Figure 3B), with small variation in the molar ellipticity and wavelength of the peaks that are due to DNA helicity and base stacking.

### Electrospray Mass Spectrometry Experiments

Although Pt-based drugs have DNA as their major target, these drugs can also bind to proteins.^15,16^ For this reason, the interaction between I-picoplatin and proteins was investigated here by ESI-MS and X-ray crystallography using protein model systems. The results were compared to analogous data obtained with cisplatin and picoplatin. ESI-MS experiments were carried out using a well-established protocol developed by the group of the University of Florence. This procedure, described in detail in several publications,^20,29,47^ involves the incubation of proteins with I-picoplatin and subsequent analysis of the resulting adducts by ESI-MS after 24 and 48 h of incubation.

**Figure 4:**
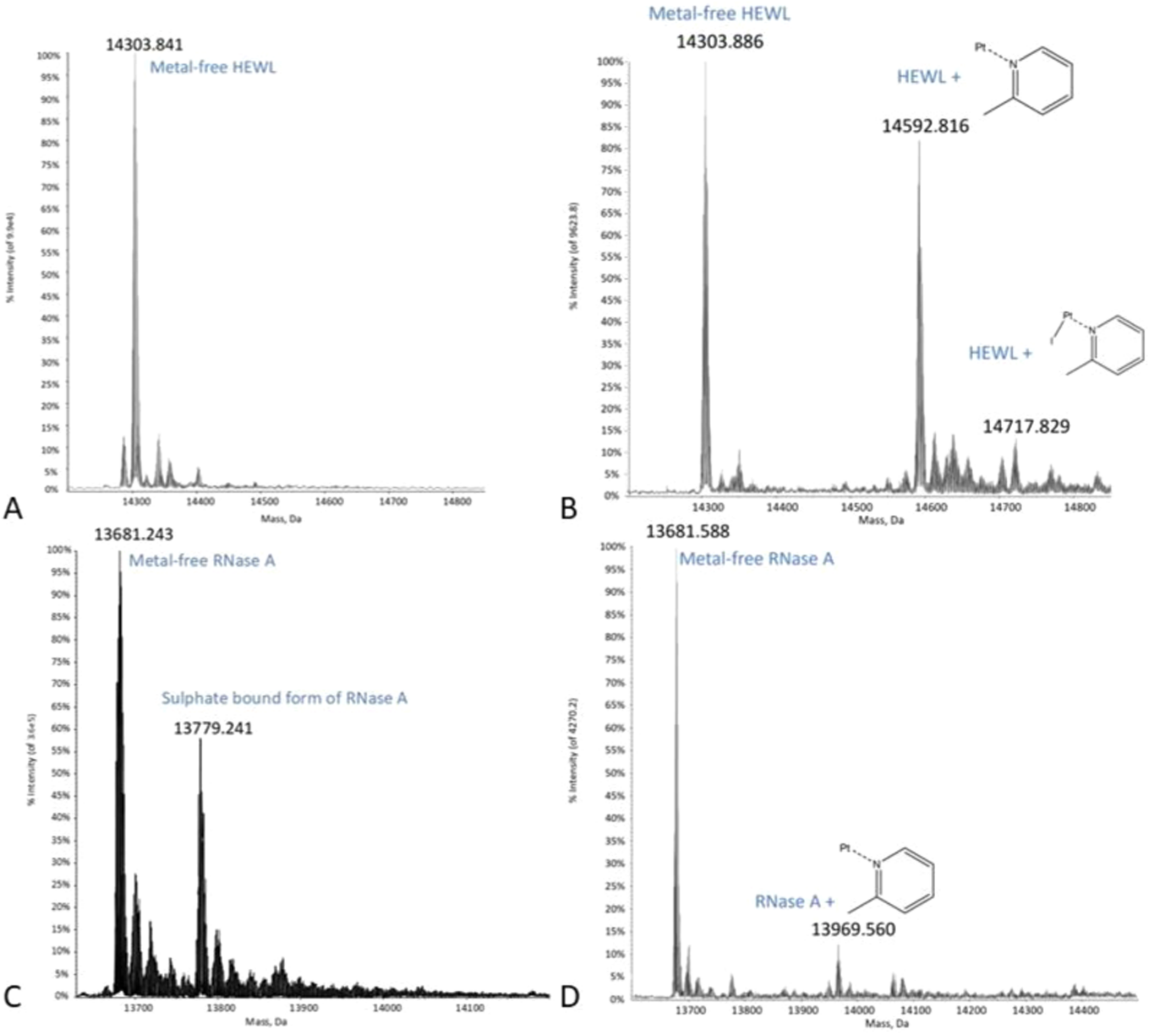
Deconvoluted spectra of (A) metal-free HEWL and (B) HEWL in the presence of I-picoplatin. (C) Metal-free RNase A and (D) RNase A in the presence of I-picoplatin. To register spectra in panels B and D, the two proteins were incubated with I-picoplatin at 37 °C for 24 h in a 1:3 protein-to-Pt molar ratio.

Figure 4 presents a comparative analysis of the ESI-MS spectra of I-picoplatin interacting with RNase A and HEWL after 24 h incubation.

The spectra corresponding to adduct formation after 48 h are shown in Figure S2. Attempts to obtain spectra of HSA in the presence of I-picoplatin failed, but given the complexity of the system, this does not indicate that HSA does not bind to the Pt compound. In all spectra collected in the presence of I-picoplatin, the peak of the metal-free protein persists, indicating that HEWL and RNase A were incompletely metalated by I-picoplatin. Interestingly, adduct formation was clearly observed when HEWL was reacted with the metal complex (Figure 4B). In fact, in the deconvoluted spectrum, two distinct signals at mass values higher than the 14303 Da signal, belonging to the metal-free protein (Figure 4A), were found. In particular, a signal was observed at 14592 Da (shift = +288 Da), which corresponds well to the adduct of HEWL with the [Pt(2-methylpyridine)]^2+^ fragment, and a signal was found at 14721 Da (shift = +415 Da), which can be attributed to the formation of the protein adduct bearing the [PtI(2-methylpyridine)]^+^ fragment. The mass spectrum of metal-free RNase A is characterized by prominent peaks at 13681 and 13779 Da, attributed to the protein and its sulfate-bound form, respectively (Figure 4C). The interaction of RNase A with I-picoplatin results in a reaction pattern similar to those reported for HEWL (Figure 4D), with the metal-free protein signal at 13680 Da and a new signal at 13969 Da, indicating a 1:1 binding of the protein with the [Pt(2-methylpyridine)]^2+^ fragment.

Overall, these results support the conclusion that I-picoplatin, when reacting with these two proteins, preserves the 2-methylpyridine ligand, consistent with previous observations for picoplatin.^27^ One iodide ligand can also be conserved. This is rather surprising considering that it has been shown that in solution, I-picoplatin releases its 2-methylpyridine ligand.^28^

### Structures of the Adduct with HEWL

The structure of the HEWL adduct with I-picoplatin has been obtained under three different experimental conditions: 1.1 M NaCl and 0.1 M sodium acetate buffer pH 4.0 (structure **A**), 0.8 M succinic acid pH 7.0 (structure **B**), and 2.0 M sodium formate and 0.1 M Hepes buffer pH 7.5 (structure **C**). Unlike the structures of HEWL with picoplatin, where two different approaches were chosen to produce crystals of the adducts,^27^ in this case a single method was used. The drop containing protein crystals was saturated with I-picoplatin powder. In this way, the metal complex slowly dissolves and diffuses through crystal solvent channels reaching the protein binding sites. In all the structures the analysis of the Fourier difference e. d. maps suggests that I-picoplatin reacts with the protein and Pt-containing fragments bind the side chains of different residues with an occupancy <1.0. Due to the limited occupancy and possible conformational disorder, ligands coordinating the metal have not been modeled in all the Pt binding sites. The three structures are refined at 1.48–2.25 Å resolution range. Cα root-mean-square deviations (rmsd) from the metal-free protein structure (PDB entry 193L^37^) is within the range 0.24–0.34 Å. This indicates that the overall conformation of the protein remains unchanged upon interaction with the platinum compound.

**Figure 5:**
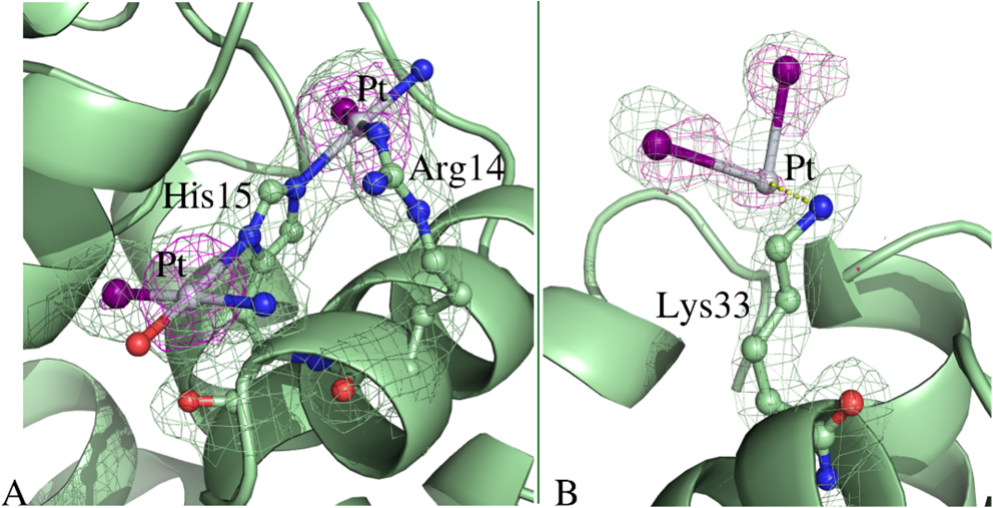
Pt binding sites in HEWL structure **A**. (A) A Pt fragment binds the ND1 atom of His15; a second Pt center binds the NE2 atom of His15 and is close to one of N atoms of guanidinium group of Arg14. (B) Noncovalent binding of a Pt fragment close to Lys33 is shown. 2Fo-Fc e.d. maps are contoured at 1.0σ (pale green); anomalous difference e.d. map is contoured at 3.0σ (magenta).

Structure **A** (Figure S3A) refines to *R*-factor/*R*_free_ values of 0.206/0.269. Three Pt-containing fragments have been found in this structure: Pt binds the side chain of His15 (Figure 5A) and is close to Arg14 (Figure 5A); an additional Pt-containing fragment is found not far from the side chain of Lys33 (Figure 5B). The side chain of His15 has been frequently identified as Pt binding site in the HEWL adducts of platinum complexes, including picoplatin,^27^ cisplatin and its diiodido analog.^15,29,48−50^ The Pt-containing fragment coordinated to the side chain of His15 retains one iodide ligand, as suggested by the anomalous ed map (Figure 5A). The other two ligands have been interpreted as a water molecule and an ammonia ligand, but from crystallographic data, we cannot exclude that ammonia ligands are replaced by water molecules. The other Pt center coordinated to His15 is close to atoms of the Arg14 side chain and completes its coordination sphere with one iodide and one ammonia ligand (or a water molecule). At both sites, the square planar geometry of Pt is retained.

Close to the side chain of Lys33 (Pt•••NZ atoms distance= 3.97 Å), the e. d. map is not clear enough to model all Pt ligands. However, at this site, it seems that two iodides are bound to the metal center (Figure 5B).

Three binding sites were identified also in structure **B** (Figure S3B), which refines to *R*-factor/*R*_free_ values of 0.227/0.259 and is obtained at pH 7.0. The first one is close to the side chain of His15, where an iodide and a water molecule/ammonia ligand were modeled close to the metal center. An unassigned ligand would complete the Pt coordination sphere (Figure S4A). The same I-picoplatin fragment is found close to the side chain of Arg14, which adopts two alternative conformations in the structure (Figure S4B). The third Pt-containing fragment interacts with the protein, as in structure **A**, not far from the side chain of Lys33 (Figure S4C).

Different results have been obtained in Structure **C** (Figure S3C), which refines to *R*-factor/*R*_free_ values of 0.227/0.277. In this structure, obtained at pH 7.5, the analysis of e. d. maps suggests the presence of Pt atoms close to side chains of His15 (Figure S5A), and close to both Arg14 and His15 (Figure S5A), as in Structure **A** (Figure 5A), but also the presence of an additional Pt binding site at the N-terminus (Figure S5B). In all of these sites, the Pt coordination sphere is completed by water molecules and/or ammonia ligands. Thus, in this structure iodide ligands are not observed.

### Structures of the Adduct with RNase A

RNase A crystals with two protein chains in the asymmetric unit were used to produce crystals of the protein adduct with I-picoplatin. These crystals, which were obtained in the presence of 30% DMSO, diffract X-rays at 1.78 Å resolution. The X-ray structure refines to *R*-factor/*R*_free_ values of 0.211/0.276. The RNase A structure has a V-shaped motif and comprises two subdomains defined by two antiparallel β-sheets, termed V1 (residues 61–63, 71–75, 105–111, and 116–124) and V2 (residues 42–46, 82–87, and 96–101), which are linked by a hinge region constituted by residues 47–48, 80–81, and 102–104.^45^ The V-shaped motif of the protein in these crystals is not significantly affected by Pt-containing fragment binding (Figure S6). Cα rmsds from the structure of the metal-free protein (PDB entry 1JVT(36)) are within the range 0.27–0.52 Å. The behavior of I-picoplatin upon reaction with RNase A is comparable to that observed with HEWL. In fact, the analysis of the structures shows that different Pt-containing fragments ([PtI_2_(OH_2_)], [PtI(OH_2_)_2_]^+^, [PtI(OH_2_)X]^+^ (X = undefined but not I)) coordinate residue side chains. In these fragments, one of the water molecules could be an ammonia ligand.

**Figure 6:**
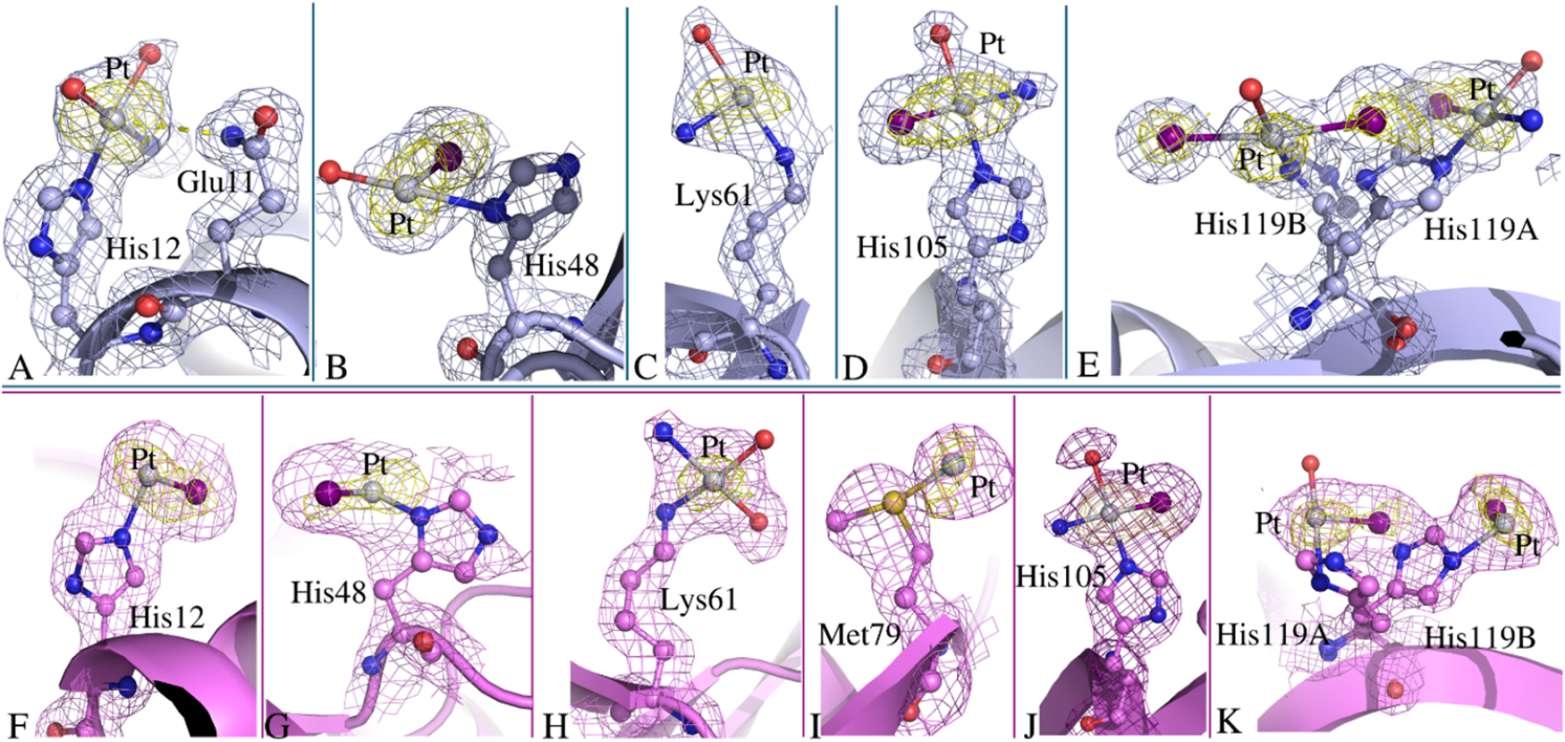
Pt binding sites in molecules A (light blue) and B (violet) of the adduct of RNase A with I-picoplatin. Pt binds close to (A) His12, (B) His48, (C) Lys61, (D) His105, (E) His119 (the side chain of His119 adopts two different conformations) of molecule A, and close to (F) His12, (G) His48, (H) Lys61, (I) Met79, (J) His105, (K) His119 (the side chain of His119 adopts two different conformations) of molecule B. 2Fo-Fc e.d. maps are contoured at 1.0σ (light blue and violet); anomalous difference e.d. map is contoured at 3.0σ (yellow).

The RNase A binding sites of I-picoplatin are not dissimilar from those observed when picoplatin binds the protein under the same experimental conditions. I-picoplatin fragments coordinate almost to the same residues of the protein bound to picoplatin moieties:^27^ the side chains of His12, His48, Lys61, His105, and His119 of both RNase A molecules in the asymmetric unit and the side chain of Met79 of molecule B (Figure 6). Lys61 is the only Pt binding identified in the structure of the adduct with I-picoplatin that is not observed in the structure of RNase A with picoplatin. Notably, close to His119 of molecule A, a [PtI_2_(OH_2_)] fragment is found, with the I ligands trans to each other. This finding supports previous data demonstrating that I-picoplatin is able to isomerize in solution.^28^

### Structures of the Adduct with HSA

HSA is the most abundant protein in the cardiovascular system. It is able to bind a large variety of molecules, including metallodrugs, affecting their distribution and efficacy.^17^ Despite intensive efforts have been devoted to the identification of the details of the interaction between metallodrugs and this protein, structural bases of Pt-based drugs binding to HSA remain elusive.^47^ In this respect, it should be noted that structural data on adducts formed upon reaction of HSA with Pt-based drugs are still very rare: the only known structures of platinated HSA are those with cisplatin in the absence^32,33^ and presence of fatty acids.^33^

**Figure 7:**
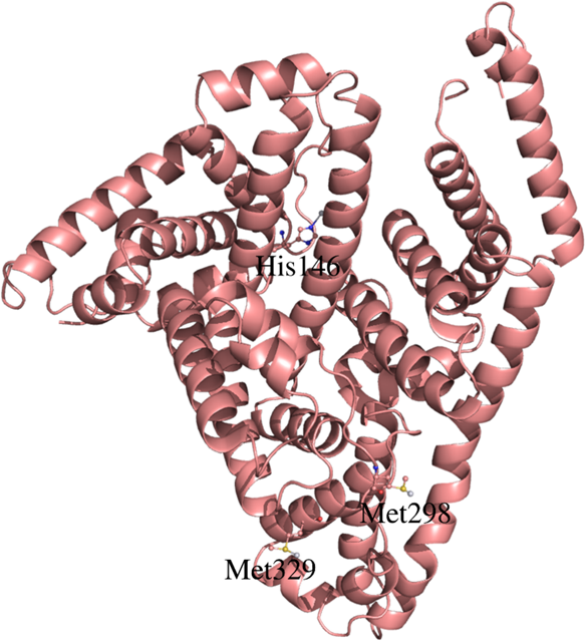
Overall structure of the adduct formed upon reaction of HSA with I-picoplatin. His146, Met298, and Met329 are highlighted.

Literature data demonstrated that picoplatin binds HSA,^32^ however details on the protein sites involved in the recognition of the Pt-drug have not been reported until now. Here, we have studied the reactivity of HSA with I-picoplatin, solving two low-resolution structures of the I-picoplatin/HSA adduct. Given the similarity of the two structures, only that at higher resolution is here described. The HSA structure in its adduct with I-picoplatin has been refined to a resolution of 3.9 Å (Figure 7). The final model, which lacks four residues at the N-terminal tail, residues of loops 76–89 and 501–513 and a few residues at the C-terminal tail, refines to a crystallographic R-factor of 0.293 (*R*_free_ = 0.327). The platinated HSA structure is almost identical to that in the absence of the metal compound: the protein retains its heart-shape due to the assembly of three helical domains (labeled I–III), each of which is divided into two subdomains (A and B) and to the presence of 17 disulfide bridges. RMSD from the starting model with PDB code 4S1Y(32) is as low as 0.04 Å, but this is not surprising considering that the structure has been refined using restraints.

**Figure 8:**
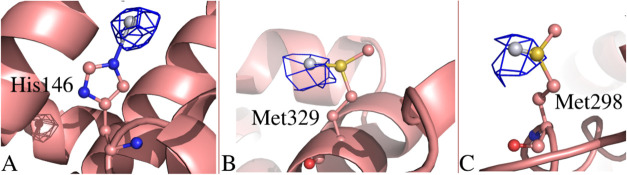
Details of the Pt binding sites in platinated HSA. Pt binds close to (A) His146, (B) Met329, and (C) Met298. Anomalous difference e.d. map of these sites is contoured at 3.0σ (blue).

Although the structure has been determined at low resolution, it shows unambiguous evidence of Pt binding sites (Figure 8). A total of three Pt binding sites were clearly identified in the Fourier difference and anomalous difference e. d. maps. Pt is found close to the side chains of His146, Met298, and Met329 (Figure 8). Occupancy of Pt centers have been fixed at 0.50; *B*-factors of metal centers are 72.3, 82.9, and 78.7 Å^2^, respectively, in line with the *B*-factor values of coordinating residue atoms. Met298 and Met329 are cisplatin binding sites both in the absence and presence of fatty acids, while His146 was believed to be a cisplatin binding site only in the presence of fatty acids up to now.^47^

## Conclusions

The cytotoxicity of I-picoplatin, picoplatin, and cisplatin was tested on selected human cancer cell lines with different sensitivity to cisplatin. I-picoplatin is more effective than picoplatin and, in most lines, also than cisplatin against the cancer cells tested. Notably, I-picoplatin is significantly more toxic than cisplatin and picoplatin on HeLa and A2780R lines with intrinsic and acquired resistances to cisplatin, respectively. I-picoplatin also exhibited some selectivity for tumor versus noncancerous cells (Table 1). Differences were observed for I-picoplatin and picoplatin in the case of the cell cycle modification of HeLa cells. The results obtained from testing the selected platinum compounds at the cellular level suggest that I-picoplatin has an altered mechanism of action compared to that of cisplatin and picoplatin, which may involve additional interactions with biomacromolecules. The binding of I-picoplatin to DNA was investigated by using EB displacement fluorescence assay and CD. To investigate its reactivity with proteins, we have collected ESI-MS spectra and refined the X-ray structure of the adducts formed with HEWL and RNase A under the same experimental conditions used to study the reaction with cisplatin and picoplatin.^27^ Recently, picoplatin has been shown to bind HEWL and RNase A, forming adducts with Pt-containing fragments attached to His and Asp side chains.^27^ In solution, studies have shown that I-picoplatin behaves differently to picoplatin.^28^ The present data show that the iodinated derivative can retain the 2-methylpyridine ligand and that Pt-containing fragments, which can retain the iodide ligands, coordinate the side chains of His15, Arg14/His15, and the N-terminus in the adduct with HEWL and the side chains of His12, His48, His105, and His119 in the reaction with RNase A, similar to picoplatin. However, the data also show differences in the reactivity of I-picoplatin with proteins compared with that of the parent drug. Indeed, in the metal/protein adducts, I-picoplatin fragments with I ligands in *trans* to each other are also found bound to the protein residues. This finding may be related to the different behavior of picoplatin and I-picoplatin in solution^28^ and to the differences in the activity of the two drugs.

Notably, the work also provides a rare example of the structure of the adduct formed when a Pt-based compound reacts with HSA.^47^ Although solved at low resolution, the new structure of platinated HSA clearly shows that the metal compound binds the side chains of His146, Met298, and Met329, suggesting that the compound reacts differently with the protein compared to cisplatin.^32,33^

## Supporting Information Available

Additional experimental details, materials, and methods, including figures and tables (PDF)

## Abbreviations

HEWL: hen egg white lysozyme
HSA: human serum albumin
A2780 and A2780R: human ovarian carcinoma (cisplatin-sensitive and -resistant, respectively)
HeLa: cervical carcinoma
OE33: esophageal carcinoma
MDA-MB-231: triple-negative breast carcinoma
MRC-5 pd30: cells derived from normal human lung tissue
DMF: *N*,*N*′-dimethylformamide
GSH: glutathione
NMR: nuclear magnetic resonance
ESI-MS: electrospray ionization mass spectrometry

## References

[ref1] Bodor J. N.; Kasireddy V.; Borghaei H. First-Line Therapies for Metastatic Lung Adenocarcinoma Without a Driver Mutation. J. Oncol. Pract. 2018, 14 (9), 529–535. 10.1200/JOP.18.00250. 30205771

[ref2] Rixe O.; Ortuzar W.; Alvarez M.; Parker R.; Reed E.; Paull K.; Fojo T. Oxaliplatin, Tetraplatin, Cisplatin, and Carboplatin: Spectrum of Activity in Drug-Resistant Cell Lines and in the Cell Lines of the National Cancer Institute’s Anticancer Drug Screen Panel. Biochem. Pharmacol. 1996, 52 (12), 1855–1865. 10.1016/S0006-2952(97)81490-6. 8951344

[ref3] Yusoh N. A.; Ahmad H.; Gill M. R. Combining PARP Inhibition with Platinum, Ruthenium or Gold Complexes for Cancer Therapy. ChemMedChem 2020, 15 (22), 2121–2135. 10.1002/cmdc.202000391. 32812709 PMC7754470

[ref4] Huang D.; Savage S. R.; Calinawan A. P.; Lin C.; Zhang B.; Wang P.; Starr T. K.; Birrer M. J.; Paulovich A. G. A Highly Annotated Database of Genes Associated with Platinum Resistance in Cancer. Oncogene 2021, 40 (46), 6395–6405. 10.1038/s41388-021-02055-2. 34645978 PMC8602037

[ref5] Dasari S.; Bernard Tchounwou P. Cisplatin in Cancer Therapy: Molecular Mechanisms of Action. Eur. J. Pharmacol. 2014, 740, 364–378. 10.1016/j.ejphar.2014.07.025. 25058905 PMC4146684

[ref6] Notaro A.; Gasser G. Monomeric and Dimeric Coordinatively Saturated and Substitutionally Inert Ru(ii) Polypyridyl Complexes as Anticancer Drug Candidates. Chem. Soc. Rev. 2017, 46 (23), 7317–7337. 10.1039/C7CS00356K. 29027562

[ref7] Meier-Menches S. M.; Gerner C.; Berger W.; Hartinger C. G.; Keppler B. K. Structure–Activity Relationships for Ruthenium and Osmium Anticancer Agents – towards Clinical Development. Chem. Soc. Rev. 2018, 47 (3), 909–928. 10.1039/C7CS00332C. 29170783

[ref8] Franz K. J.; Metzler-Nolte N. Introduction: Metals in Medicine. Chem. Rev. 2019, 119 (2), 727–729. 10.1021/acs.chemrev.8b00685. 30990707

[ref9] Metal-Based Anticancer Agents; Casini A.; Vessières A.; Meier-Menches S. M., Eds.; The Royal Society of Chemistry, 2019.

[ref10] Treat J.; Schiller J.; Quoix E.; Mauer A.; Edelman M.; Modiano M.; Bonomi P.; Ramlau R.; Lemarie E. ZD0473 Treatment in Lung Cancer: An Overview of the Clinical Trial Results. Eur. J. Cancer 2002, 38, S13–S18. 10.1016/S0959-8049(02)80016-8. 12645908

[ref11] Raynaud F. I.; Boxall F. E.; Goddard P. M.; Valenti M.; Jones M.; Murrer B. A.; Abrams M.; Kelland L. R. Cis-Amminedichloro(2-Methylpyridine) Platinum(II) (AMD473), a Novel Sterically Hindered Platinum Complex: In Vivo Activity, Toxicology, and Pharmacokinetics in Mice. Clin. Cancer Res. 1997, 3 (11), 2063–2074. 9815598

[ref12] Sharp S. Y.; O’Neill C. F.; Rogers P.; Boxall F. E.; Kelland L. R. Retention of Activity by the New Generation Platinum Agent AMD0473 in Four Human Tumour Cell Lines Possessing Acquired Resistance to Oxaliplatin. Eur. J. Cancer 2002, 38 (17), 2309–2315. 10.1016/S0959-8049(02)00244-7. 12441268

[ref13] Tang P.; Wang J.; Bourne P. Molecular Classifications of Breast Carcinoma with Similar Terminology and Different Definitions: Are They the Same?. Hum. Pathol. 2008, 39 (4), 506–513. 10.1016/j.humpath.2007.09.005. 18289638

[ref14] Shahlaei M.; Asl S. M.; Derakhshani A.; Kurek L.; Karges J.; Macgregor R.; Saeidifar M.; Kostova I.; Saboury A. A. Platinum-Based Drugs in Cancer Treatment: Expanding Horizons and Overcoming Resistance. J. Mol. Struct. 2024, 1301, 137366 10.1016/j.molstruc.2023.137366.

[ref15] Ferraro G.; Loreto D.; Merlino A. Interaction of Platinum-Based Drugs with Proteins: An Overview of Representative Crystallographic Studies. Curr. Top. Med. Chem. 2021, 21 (1), 6–27. 10.2174/1568026620666200624162213. 32579504

[ref16] Messori L.; Merlino A. Cisplatin Binding to Proteins: A Structural Perspective. Coord. Chem. Rev. 2016, 315, 67–89. 10.1016/j.ccr.2016.01.010.

[ref17] Pinato O.; Musetti C.; Sissi C. Pt-Based Drugs: The Spotlight Will Be on Proteins. Metallomics 2014, 6 (3), 380–395. 10.1039/C3MT00357D. 24510227

[ref18] Pinato O.; Musetti C.; Farrell N. P.; Sissi C. Platinum-Based Drugs and Proteins: Reactivity and Relevance to DNA Adduct Formation. J. Inorg. Biochem. 2013, 122, 27–37. 10.1016/j.jinorgbio.2013.01.007. 23435290 PMC3602126

[ref19] Merlino A. Recent Advances in Protein Metalation: Structural Studies. Chem. Commun. 2021, 57 (11), 1295–1307. 10.1039/D0CC08053E. 33464256

[ref20] Merlino A.; Marzo T.; Messori L. Protein Metalation by Anticancer Metallodrugs: A Joint ESI MS and XRD Investigative Strategy. Chem.—Eur. J. 2017, 23 (29), 6942–6947. 10.1002/chem.201605801. 28071831

[ref21] Tanley S. W. M.; Schreurs A. M. M.; Kroon-Batenburg L. M. J.; Meredith J.; Prendergast R.; Walsh D.; Bryant P.; Levy C.; Helliwell J. R. Structural Studies of the Effect That Dimethyl Sulfoxide (DMSO) Has on Cisplatin and Carboplatin Binding to Histidine in a Protein. Acta Crystallogr., Sect. D 2012, 68 (5), 601–612. 10.1107/S0907444912006907. 22525758

[ref22] Messori L.; Merlino A. Cisplatin Binding to Proteins: Molecular Structure of the Ribonuclease A Adduct. Inorg. Chem. 2014, 53 (8), 3929–3931. 10.1021/ic500360f. 24694179

[ref23] Helliwell J. R.; Tanley S. W. M. The Crystal Structure Analysis of the Relative Binding of Cisplatin and Carboplatin in a Mixture with Histidine in a Protein Studied at 100 and 300 K with Repeated X-Ray Irradiation. Acta Crystallogr., Sect. D 2013, 69 (1), 121–125. 10.1107/S090744491204423X. 23275170

[ref24] Picone D.; Donnarumma F.; Ferraro G.; Russo Krauss I.; Fagagnini A.; Gotte G.; Merlino A. Platinated Oligomers of Bovine Pancreatic Ribonuclease: Structure and Stability. J. Inorg. Biochem. 2015, 146, 37–43. 10.1016/j.jinorgbio.2015.02.011. 25756333

[ref25] Ferraro G.; Pica A.; Russo Krauss I.; Pane F.; Amoresano A.; Merlino A. Effect of Temperature on the Interaction of Cisplatin with the Model Protein Hen Egg White Lysozyme. J. Biol. Inorg. Chem. 2016, 21 (4), 433–442. 10.1007/s00775-016-1352-0. 27040953

[ref26] Messori L.; Marzo T.; Gabbiani C.; Valdes A. A.; Quiroga A. G.; Merlino A. Peculiar Features in the Crystal Structure of the Adduct Formed between *Cis* -PtI _2_ (NH_3_)_2_ and Hen Egg White Lysozyme. Inorg. Chem. 2013, 52 (24), 13827–13829. 10.1021/ic402611m. 24256441

[ref27] Ferraro G.; Lyčková T.; Massai L.; Štarha P.; Messori L.; Merlino A. Picoplatin Binding to Proteins: X-Ray Structures and Mass Spectrometry Data on the Adducts with Lysozyme and Ribonuclease A. Dalton Trans. 2024, 53 (20), 8535–8540. 10.1039/D4DT00773E. 38727007

[ref28] Štarha P.; Drahoš B.; Herchel R. An Unexpected In-Solution Instability of Diiodido Analogue of Picoplatin Complicates Its Biological Characterization. Dalton Trans. 2021, 50 (18), 6071–6075. 10.1039/D1DT00740H. 33913454

[ref29] Tanley S. W. M.; Schreurs A. M. M.; Kroon-Batenburg L. M. J.; Helliwell J. R. Room-Temperature X-Ray Diffraction Studies of Cisplatin and Carboplatin Binding to His15 of HEWL after Prolonged Chemical Exposure. Acta Crystallogr., Sect. F 2012, 68 (11), 1300–1306. 10.1107/S1744309112042005. PMC3515368 23143236

[ref30] Tanley S. W. M.; Schreurs A. M. M.; Kroon-Batenburg L. M. J.; Helliwell J. R. Re-Refinement of 4g4a: Room-Temperature X-Ray Diffraction Study of Cisplatin and Its Binding to His15 of HEWL after 14 Months Chemical Exposure in the Presence of DMSO. Acta Crystallogr., Sect. F 2016, 72 (3), 253–254. 10.1107/S2053230X16000856. PMC4774887 26948967

[ref31] Tanley S. W. M.; Helliwell J. R. Chemical Conversion of Cisplatin and Carboplatin with Histidine in a Model Protein Crystallized under Sodium Iodide Conditions. Acta Crystallogr., Sect. F 2014, 70 (9), 1127–1131. 10.1107/S2053230X14013995. PMC4157406 25195879

[ref32] Ferraro G.; Massai L.; Messori L.; Merlino A. Cisplatin Binding to Human Serum Albumin: A Structural Study. Chem. Commun. 2015, 51 (46), 9436–9439. 10.1039/C5CC01751C. 25873085

[ref33] Chen S.; Yuan C.; Jiang L.; Luo Z.; Huang M. Crystallographic Analysis of Interaction between Cisplatin and Human Serum Albumin: Effect of Fatty Acid. Int. J. Biol. Macromol. 2022, 216, 172–178. 10.1016/j.ijbiomac.2022.06.181. 35788007

[ref34] Vonrhein C.; Flensburg C.; Keller P.; Sharff A.; Smart O.; Paciorek W.; Womack T.; Bricogne G. Data Processing and Analysis with the *autoPROC* Toolbox. Acta Crystallogr., Sect. D 2011, 67 (4), 293–302. 10.1107/S0907444911007773. 21460447 PMC3069744

[ref35] McCoy A. J.; Grosse-Kunstleve R. W.; Adams P. D.; Winn M. D.; Storoni L. C.; Read R. J. *Phaser* Crystallographic Software. J. Appl. Crystallogr. 2007, 40 (4), 658–674. 10.1107/S0021889807021206. 19461840 PMC2483472

[ref36] Vitagliano L.; Merlino A.; Zagari A.; Mazzarella L. Reversible Substrate-induced Domain Motions in Ribonuclease A. Proteins 2002, 46 (1), 97–104. 10.1002/prot.10033. 11746706

[ref37] Vaney M. C.; Maignan S.; Riès-Kautt M.; Ducruix A. High-Resolution Structure (1.33 Å) of a HEW Lysozyme Tetragonal Crystal Grown in the APCF Apparatus. Data and Structural Comparison with a Crystal Grown under Microgravity from SpaceHab-01 Mission. Acta Crystallogr., Sect. D 1996, 52 (3), 505–517. 10.1107/S090744499501674X. 15299672

[ref38] Murshudov G. N.; Skubák P.; Lebedev A. A.; Pannu N. S.; Steiner R. A.; Nicholls R. A.; Winn M. D.; Long F.; Vagin A. A. *REFMAC* 5 for the Refinement of Macromolecular Crystal Structures. Acta Crystallogr., Sect. D 2011, 67 (4), 355–367. 10.1107/S0907444911001314. 21460454 PMC3069751

[ref39] Nicholls R. A.; Fischer M.; McNicholas S.; Murshudov G. N. Conformation-Independent Structural Comparison of Macromolecules with *ProSMART*. Acta Crystallogr., Sect. D 2014, 70 (9), 2487–2499. 10.1107/S1399004714016241. 25195761 PMC4157452

[ref40] Emsley P.; Lohkamp B.; Scott W. G.; Cowtan K. Features and Development of *Coot*. Acta Crystallogr., Sect. D 2010, 66 (4), 486–501. 10.1107/S0907444910007493. 20383002 PMC2852313

[ref41] Holford J.; Sharp S.; Murrer B.; Abrams M.; Kelland L. In Vitro Circumvention of Cisplatin Resistance by the Novel Sterically Hindered Platinum Complex AMD473. Br. J. Cancer 1998, 77 (3), 366–373. 10.1038/bjc.1998.59. 9472630 PMC2151285

[ref42] Kelland L. Broadening the Clinical Use of Platinum Drug–Based Chemotherapy with New Analogues: Satraplatin and Picoplatin. Expert Opin. Invest. Drugs 2007, 16 (7), 1009–1021. 10.1517/13543784.16.7.1009. 17594186

[ref43] Siddik Z. H. Cisplatin: Mode of Cytotoxic Action and Molecular Basis of Resistance. Oncogene 2003, 22 (47), 7265–7279. 10.1038/sj.onc.1206933. 14576837

[ref44] Intini F. P.; Zajac J.; Novohradsky V.; Saltarella T.; Pacifico C.; Brabec V.; Natile G.; Kasparkova J. Novel Antitumor Platinum(II) Conjugates Containing the Nonsteroidal Anti-Inflammatory Agent Diclofenac: Synthesis and Dual Mechanisms of Antiproliferative Effects. Inorg. Chem. 2017, 56 (3), 1483–1497. 10.1021/acs.inorgchem.6b02553. 28102676

[ref45] Cheung-Ong K.; Giaever G.; Nislow C. DNA-Damaging Agents in Cancer Chemotherapy: Serendipity and Chemical Biology. Chem. Biol. 2013, 20 (5), 648–659. 10.1016/j.chembiol.2013.04.007. 23706631

[ref46] Sharma S.; Shah N. A.; Joiner A. M.; Roberts K. H.; Canman C. E. DNA Polymerase ζ Is a Major Determinant of Resistance to Platinum-Based Chemotherapeutic Agents. Mol. Pharmacol. 2012, 81 (6), 778–787. 10.1124/mol.111.076828. 22387291 PMC3362893

[ref47] Merlino A. Metallodrug Binding to Serum Albumin: Lessons from Biophysical and Structural Studies. Coord. Chem. Rev. 2023, 480, 215026 10.1016/j.ccr.2023.215026.

[ref48] Tanley S. W. M.; Diederichs K.; Kroon-Batenburg L. M. J.; Levy C.; Schreurs A. M. M.; Helliwell J. R. Carboplatin Binding to Histidine. Acta Crystallogr., Sect. F 2014, 70 (9), 1135–1142. 10.1107/S2053230X14016161. PMC4157408 25195881

[ref49] Messori L.; Merlino A. Protein Metalation by Metal-Based Drugs: X-Ray Crystallography and Mass Spectrometry Studies. Chem. Commun. 2017, 53 (85), 11622–11633. 10.1039/C7CC06442J. 29019481

[ref50] Marasco D.; Messori L.; Marzo T.; Merlino A. Oxaliplatin vs. Cisplatin: Competition Experiments on Their Binding to Lysozyme. Dalton Trans. 2015, 44 (22), 10392–10398. 10.1039/C5DT01279A. 25974859

