# Supplementary material for: Cytotoxicity and Binding to DNA, Lysozyme, Ribonuclease A, and Human Serum Albumin of the Diiodido Analog of Picoplatin
Source: Inorg Chem. 2025 May 2;64(18):8895–905. doi: 10.1021/acs.inorgchem.4c05424 (PMC12076543; doi:10.1021/acs.inorgchem.4c05424)
Supplement: Supplementary file 1 — ic4c05424_si_001.pdf [file ic4c05424_si_001.pdf]

Supplementary Information

Cytotoxicity and binding to DNA, lysozyme, ribonuclease A and human serum albumin of the diiodido analog of picoplatin

Giarita Ferraro, [#] *Jitka Pracharova,* [+] *Giovanni Gotte,* [§] *Lara Massai,* [†] *Michal Berecka,* [+] *Pavel Starha,* [‡] *Luigi Messori* [†] *and Antonello Merlino* [#]*

[#] Department of Chemical Sciences, University of Naples Federico II, Complesso univ. Monte Sant'Angelo, via Cinthia 21, 80126, Naples, Italy.
[+] Department of Biophysics, Faculty of Science, Palacký University Olomouc, Slechtitelu 27, 783 71 Olomouc, Czech Republic
[§] Department of Neuroscience, Biomedicine, and Movement Sciences, Biological Chemistry Section, University of Verona, Strada Le Grazie 8, I-37134 Verona, Italy
[†] Department of Chemistry "Ugo Schiff", University of Florence, via della Lastruccia 3-13, 50019, Sesto Fiorentino, Florence, Italy.
[‡] Department of Inorganic Chemistry, Faculty of Science, Palacký University Olomouc, 17. listopadu 1192/12, 771 46 Olomouc, Czech Republic.
*Correspondence to: Antonello Merlino, Department of Chemical Sciences, University of Naples Federico II, Complesso univ. Monte Sant'Angelo, via Cinthia 21, 80126, Naples, Italy. e-mail: antonello.merlino@unina.it

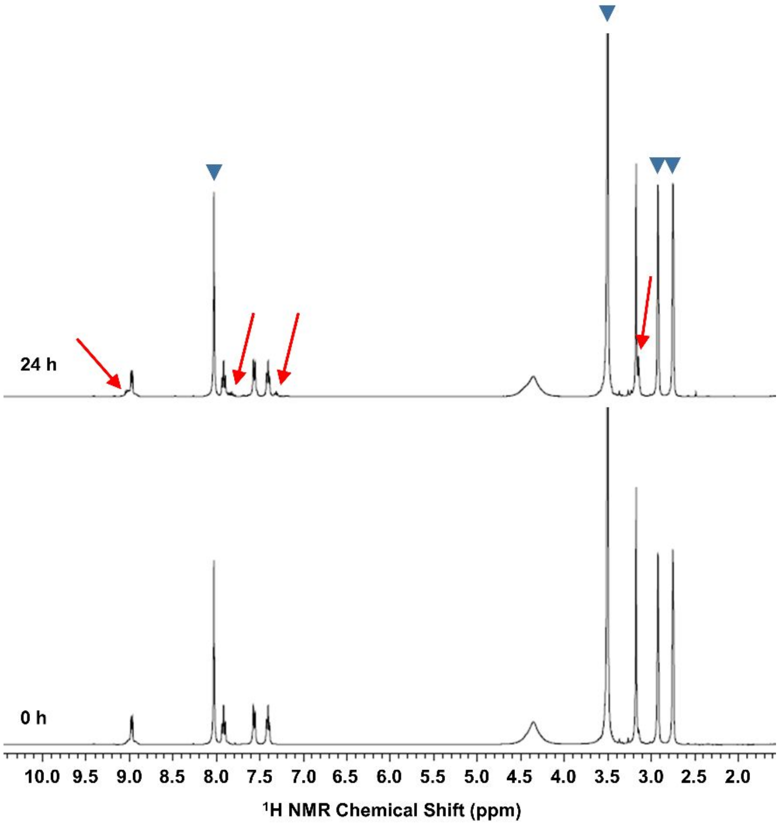

Figure S1. A part of the ^{1}H NMR spectra of the complex *cis*-[$PtI_2(NH_3)$(pic)] (I-picoplatin) at different time points (studied in DMF-d_7 and kept in the dark between the individual measurements).

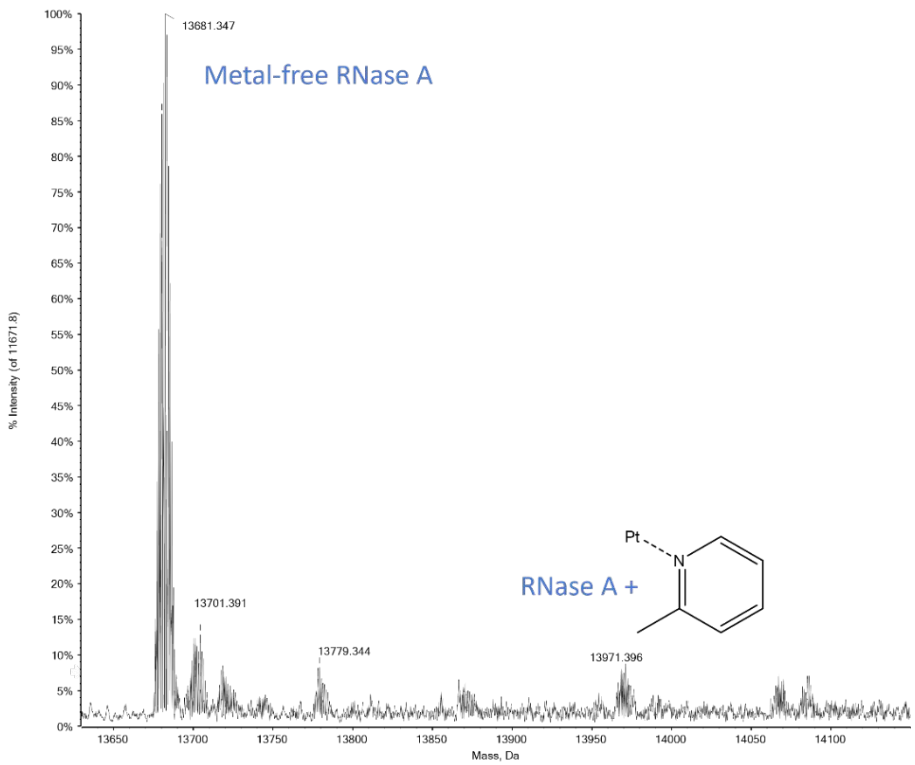

A

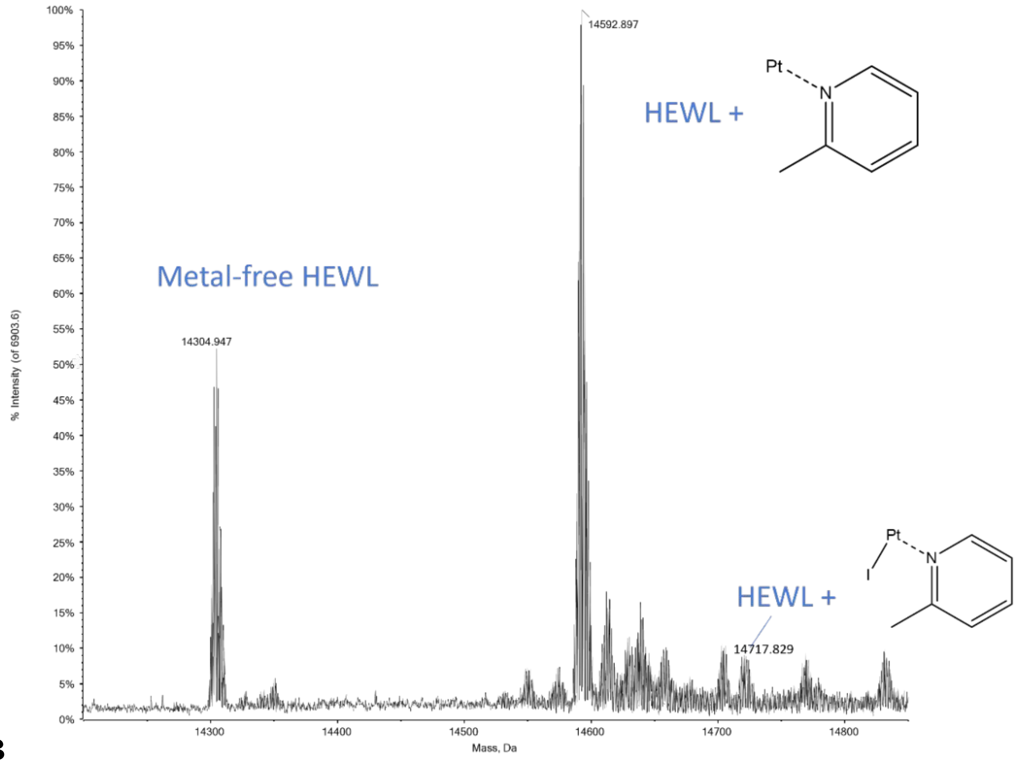

B

Figure S2. Deconvoluted spectra of A) I-picoplatin incubated with RNase A, at 37°C for 48h in 1:3 protein-to-platinum ratio; 0.1% v/v of formic acid was added just before infusion;B) I-

picoplatin incubated with HEWL, at 37°C for 48h in 1:3 protein-to-platinum ratio; 0.1% v/v of formic acid was added just before infusion.

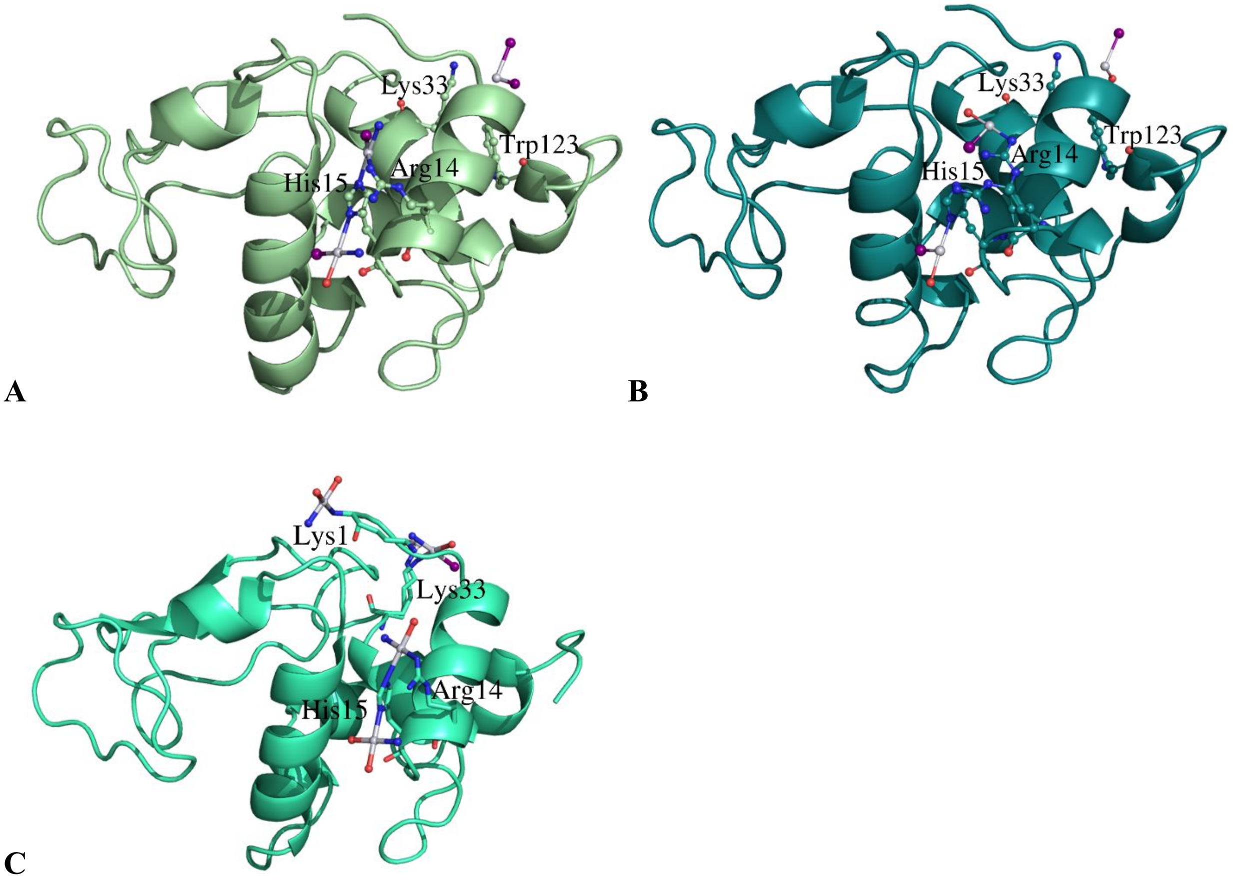

Figure S3. Overall structures of the adducts formed upon reaction of HEWL with I-picoplatin in A) 1.1 M sodium chloride, 0.1 M sodium acetate buffer pH 4.0 (structure **A**), B) 0.8 M succinic acid pH 7.0 (structure **B**) and C) 2.0 M sodium formate, 0.1 M Hepes pH 7.5 (structure **C**).

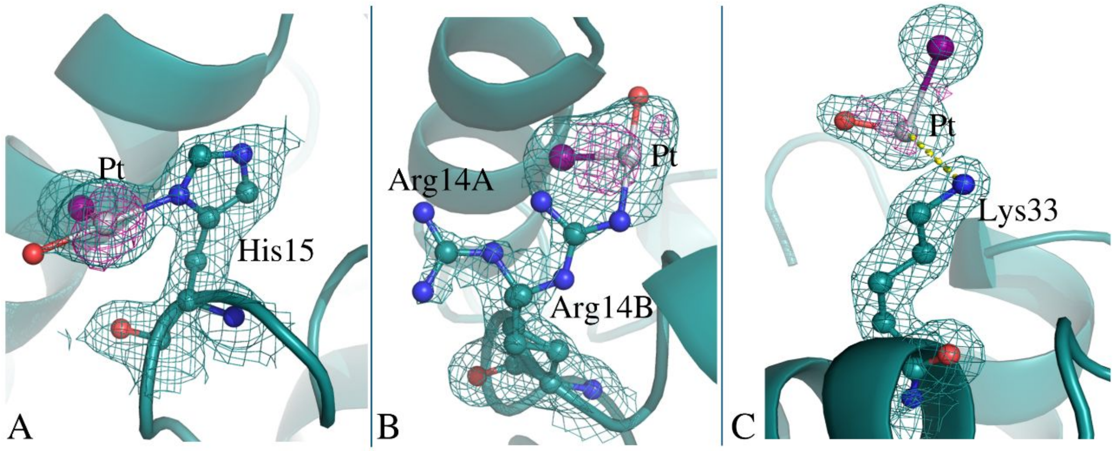

Figure S4. Details of the Pt binding sites in the HEWL structure **B**. Pt binds to the side chains of His15 (panel A) and is close to the side chain of Arg14 (panel B). An additional Pt center is observed not far from the side chain of Lys33 (panel C). 2Fo-Fc electron density maps is contoured at 1.0 σ (deepteal); anomalous difference electron density map is is contoured at 3.0 σ (magenta).

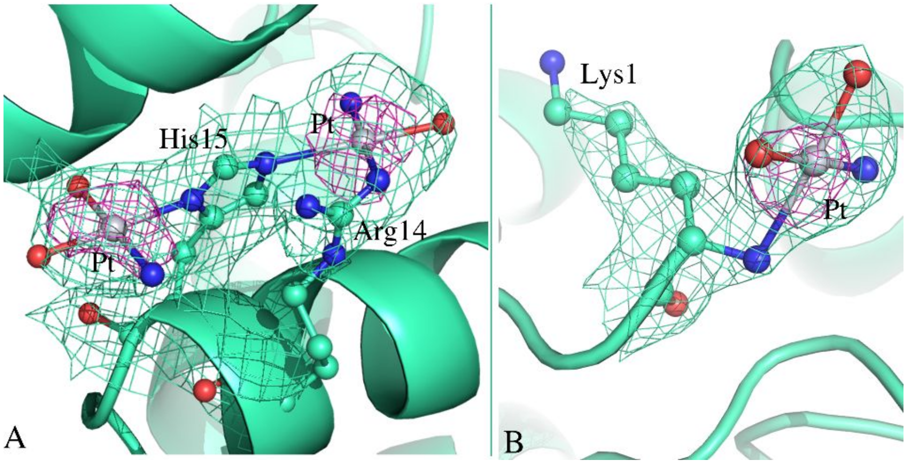

Figure S5. Details of the Pt binding sites in the HEWL structure **C**. In panel A, Pt-containing fragments are bound to His15 and is close to both Arg14 and His15 side chains. In panel B, a Pt-containing fragment binds the N atom of Lys1. 2Fo-Fc electron density maps is contoured at 1.0 σ (aquamarine); anomalous difference electron density map is is contoured at 3.0 σ (magenta).

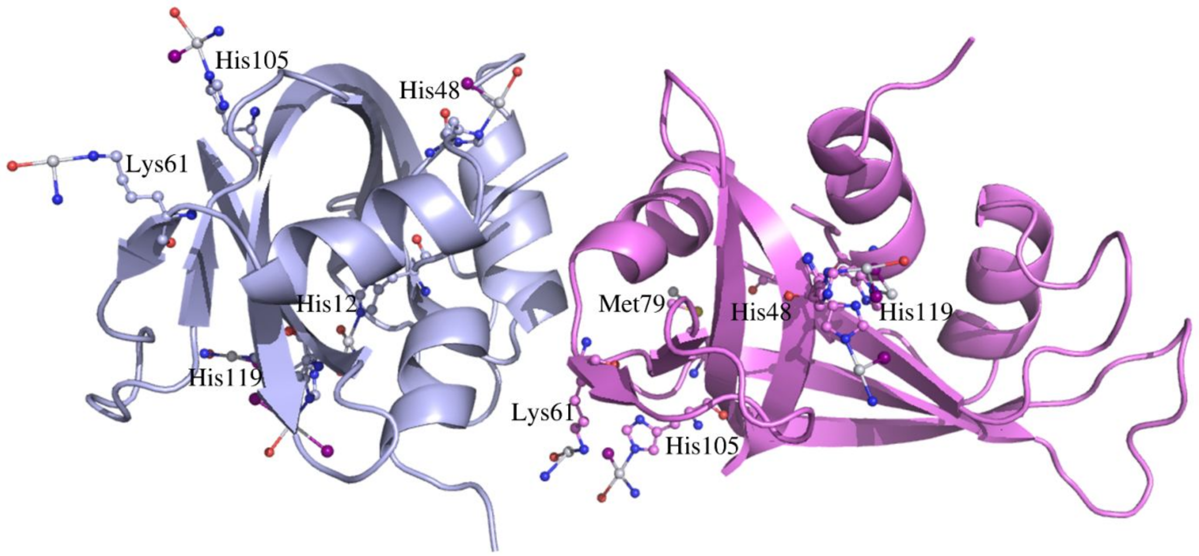

Figure S6. Overall structure of the adduct formed upon reaction of RNase A with I-picoplatin; molecules A and B of the asymmetric unit are shown in light blue and violet, respectively.

Table S1. Data collection and refinement statistics.

	*HEWL structure **A***	*HEWL structure **B***	*HEWL structure **C***	*RNase A*	*HSA*
PDB code	9HLK	9HMK	9HMQ	9HN6	9HNB
Crystallization condition	1.1 M sodium chloride and 0.1 M sodium acetate pH 4.5	0.8 M succinic acid pH 7.0	2.0 M sodium formate and 0.1 Hepes buffer pH 7.5	20% PEG4000, and 0.1 M sodium citrate pH 5.1	25−30% (w/v) PEG3350 and 50 mM potassium phosphate pH 7.5
Data collection					
Space group	$P4_32_12$	$P4_32_12$	$P4_32_12$	C2	$P12_11$
a (Å)	78.40	79.15	79.01	100.04	58.07
b (Å)	78.40	79.15	79.01	32.64	86.55
c (Å)	36.92	36.03	35.43	73.45	59.50
α (°)	90.00	90.00	90.00	90.00	90.00
β (°)	90.00	90.00	90.00	90.11	102.55
γ (°)	90.00	90.00	90.00	90.00	90.00
Resolution range (Å)	55.44-1.96 (2.00-1.96)	39.57-1.48 (1.51-1.48)	39.51-2.25 (2.28-2.25)	73.45-1.77 (1.80-1.77)	47.42-3.90 (3.97-3.90)
Observations	178798 (8885)	381283 (19781)	103377 (4761)	134865 (6936)	17727 (914)
Unique reflections	8548 (381)	19656 (975)	5691 (271)	23489 (6936)	5252 (260)
Completeness (%)	98.6 (93.4)	99.9 (100.0)	99.8 (98.0)	99.7 (100.0)	99.0 (99.6)
Redundancy	20.9 (23.3)	19.4 (20.3)	18.2 (17.6)	5.7 (5.9)	3.4 (3.5)
Rmerge (%)	0.067 (0.966)	0.075 (1.616)	0.104 (0.500)	0.077 (0.881)	0.139 (0.929)
Rmeas	0.069 (0.987)	0.077 (1.658)	0.108 (0.516)	0.085 (0.966)	0.166 (1.097)
Rpim	0.015 (0.204)	0.017 (0.366)	0.026 (0.123)	0.035 (0.393)	0.089 (0.573)
Average I/σ(I)	29.9 (4.1)	19.6 (2.3)	19.4 (9.6)	12.0 (2.4)	7.3 (2.5)
$CC_{1/2}$	0.999 (0.969)	0.999 (0.914)	0.998 (0.939)	0.997 (0.860)	0.986 (0.568)
Anom. Completeness (%)	98.5 (93.3)	100.0 (100.0)	99.2 (97.1)	98.4 (99.1)	92.0 (93.2)
Anom. Multiplicity	11.6 (12.4)	10.5 (10.7)	10.2 (9.5)	3.0 (3.0)	1.8 (1.8)
Refinement					
Resolution range (Å)	55.44-1.96	39.57-1.48	39.51-2.25	73.45-1.77	47.42-3.90
N° reflection (working set)	8124	20446	7548	1311	4073
N° non-H atoms (refinement)	1133	1193	1042	2038	4198
R-factor/R_{free}	0.206/0.269	0.227/0.259	0.227/0.277	0.211/0.276	0.293/0.327
r.m.s.d. bonds	0.011	0.009	0.008	0.009	0.009
r.m.s.d. angles (°)	1.760	1.629	1.605	1.692	1.502
Ramachandran plot					
Most favoured	106 (92.17%)	104 (92.86%)	110 (88.71%)	210 (93.75%)	458 (85.29%)
Outliers	1 (0.87%)	0 (0.00%)	3 (2.42%)	6 (2.68%)	29 (5.40%)
